# Professor Thaddeus Ulzen

**DOI:** 10.1192/bjb.2024.87

**Published:** 2025-02

**Authors:** Abdi Sanati

**Figure:**
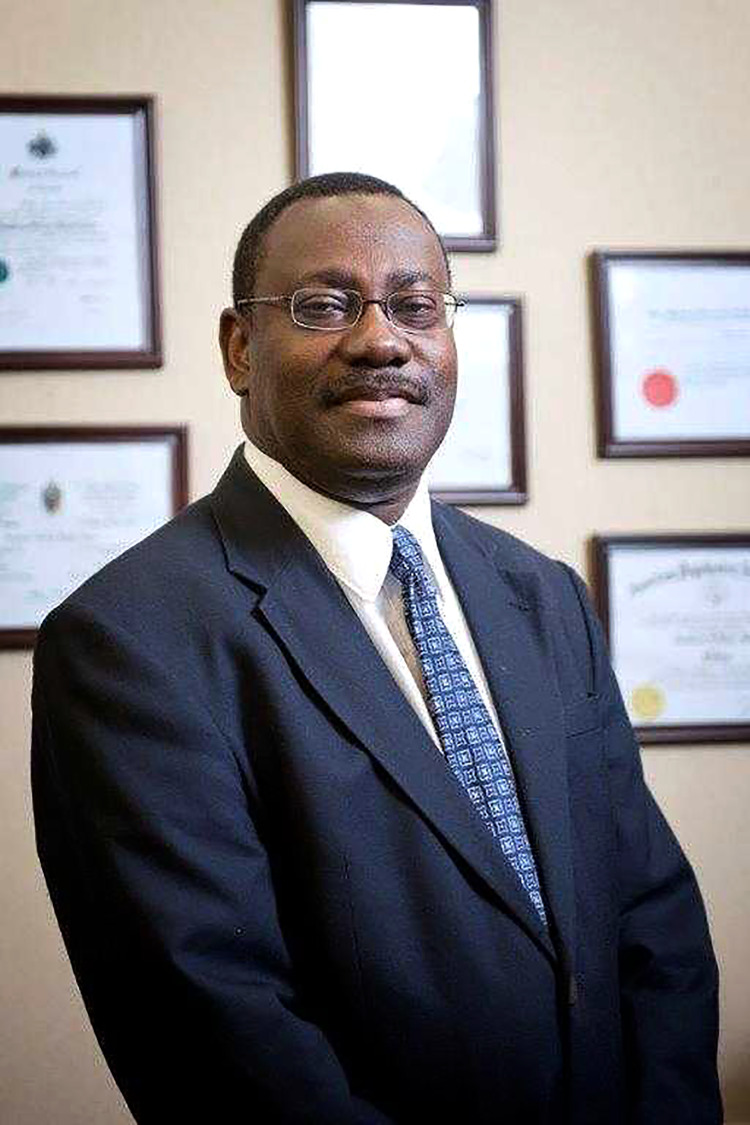

Professor Thaddeus Ulzen, FRCP(C), DFAPA, is Chair of the Department of Psychiatry and Behavioural Medicine at University of Alabama, USA. He is a child and adolescent psychiatrist with expertise in education. In 2002, Professor Ulzen was awarded the Nancy C. A. Roeske Certificate of Excellence in Medical Education by the American Psychiatric Association. I first met him in 2011, when I joined the newly founded programme for psychiatry teaching to medical students at the University of Cape Coast in Ghana. Over the years I got to know him well, and admired his sharp mind and clinical and teaching expertise. He is also an excellent writer. Professor Ulzen is someone who does not shy away from critical enquiry and seeking the truth, a quality that is becoming rarer as the years go by.

**Many thanks for your time, Professor Ulzen. Let's start with education. Could you tell us about the teaching programme that you have started in Ghana?**

Well, that is a good, broad question to start with. The teaching programme at the University of Cape Coast School of Medical Sciences was started as an invitation from the first Dean of the Medical School to provide a curriculum for psychiatry. It was at a time when in Ghana, there were probably no more than 12 psychiatrists, half of whom were already past retirement age. So, we had to figure out a good way of providing a curriculum that was designed for Ghanaian medical students, which would be delivered within that cultural context and also have longevity. These were the key issues that we were concerned about. So, I said to Professor Harold Amonoo-Kuofi, the Dean at that time, I think it was 2011, that what we could do is have a modular psychiatry curriculum, so that the teaching would take place at the same time every year for all student years. That way, I could recruit interested psychiatrists who were overseas, to form a team to come teach the course after we had designed it. That's how we started. We got interest from Ghanaian-born and other psychiatrists who were not Ghanaian, but had some familiarity with the Ghanaian environment, and also just psychiatrists who were interested in the experience of teaching in Ghana. So that's how the programme evolved.

**As you know I was one of them. And yes, it has been a great experience for me. Could you explain how you measure the success of the programme?**

There are several aspects to this. First, one would think immediately about how many students are likely to decide to go into psychiatry after graduation. That may be one metric. But really, for me, what I was more interested in was accepting the fact that the majority of the medical students were not going to be psychiatrists. So, I wanted a curriculum that would integrate psychiatry into medicine enough that the graduates of the medical school would be what I would call psychiatrically informed primary care physicians. So that they would enter the specialties of their choice, which were maybe internal medicine, paediatrics or whatever, but that they would approach their patients more eclectically, with a lens that was more biopsychosocial. This would allow them not to miss psychiatric conditions. This would give them enough of a skill set that they would not be anxious about having to handle psychiatric conditions within the framework of general medical practice. For me, that was really the goal. One of the things that we did was to collect data on attitudes to psychiatry before they entered the programme and 2 years after, at graduation. We collected some data, but quite frankly, we haven't analysed or published it yet, but these are some of metrics that we examined.

**I have to say hearing that the people that we trained are much more aware of conditions such as delirium in general hospitals was positive feedback. I was also wondering, how do you think programmes like this can inform the global teaching of psychiatry?**

I believe that a programme like this could serve as a model for other countries in Africa. Specifically, I mean, we use other terms, developing countries, Global South, whatever, but the essentially impoverished countries in Africa. Many of these countries do have trained psychiatrists and other mental health professionals who are in the Rich North, many of whom also go home for different reasons. The way we've set up this model in Cape Coast is that we have a 4-week teaching period, and people who are involved in the programme can indeed spend a week, 2 weeks or more. So, the time allocation that is needed to participate in the programme need not be more than 2 weeks every year. Because it is modular, people have advance notice so that they can plan, and this makes it easily sustainable, as the Cape Coast programme has now been running for about 12 years, with a core faculty that has remained committed to it pretty much for that period of time. A few other faculty have come and gone. The longevity has allowed us to train local psychiatry trainees, who have become psychiatrists during this period and have become teachers in this programme. So I think that the success of the programme not only has to do with the students, but also with locally trained psychiatrists who have been mentored by the more experienced teachers and have now become part of the teaching capacity within Ghana, not just for the Cape Coast Medical School, but teaching in other medical schools too.

**I have to say that last time we were there, we were told that now there are about 70 psychiatrists and soon there will be 100.**

That's right.

**And I think when they tell us that there is no need for us, then we could say that it was truly successful.**

That is right. I think when you consider that when we started the programme, as I said at the beginning, there were 12 psychiatrists in Ghana. In the period of about 12 years, there are now about 100 psychiatrists in practice or in training in postgraduate programmes within the country, to the extent that psychiatry is now no longer considered an orphan specialty by the Ghana College of Physicians and Surgeons. So, this has been quite successful. But of course, this programme has not been the only reason why there are 100 psychiatrists in Ghana right now. The students from this programme have also participated in the annual mental health debate, which was started by Professor Vincent Agyapong, currently Chair at Dalhousie University in Canada. I believe that University of Cape Coast School of Medical Sciences students have participated in the national debate more often than their peers, so that says something about our programme. There are also other activities that have taken place in other medical schools that have promoted psychiatry as a discipline. To be honest with you, I think one of the biggest aspects of this programme has been to destigmatise psychiatry as a specialty within the medical education community, that through this curriculum, we're able to highlight advances in neurosciences to make students and others understand that the science of the brain is what underpins clinical psychiatry.

**Let us move on to another topic, because that is something that I really liked. The research that you did on the bus drivers in Ghana. Could you give a short summary for my readers?**

Essentially, the study was a study of attention-deficit hyperactivity disorder (ADHD) in unionised commercial drivers. The reason I undertook this study was that in all the years that I visited Ghana, I observed many of these drivers to be extremely reckless and impulsive on the roads, so I started thinking why is this so? Then it struck me that to become a driver in Ghana and have a commercial vehicle, one of the pathways is academic failure. When somebody fails academically, then they go get a job as a driver, because they can use their hands and other skills that they might have. So, it occurred to me that it is possible that a lot of these people have failed formal education because they have untreated ADHD and that these drivers are being overrepresented in this profession, or these people were being selected into driving commercial vehicles. To cut a long story short, we got some funding from my College, a small grant. We screened the drivers for ADHD, and then we also examined a control group in the community. We found that the prevalence of ADHD in the community was consistent with worldwide prevalence of about 7%. However, we found that in the commercial drivers, it was as high as 22%. That is, one in five of these commercial drivers had untreated ADHD, and that this may be contributing significantly to road safety issues in Ghana. So again, it was a way of also bringing the relevance of psychiatry into everyday life. Again, this is part of what I call the destigmatisation of our discipline, when we're able to translate findings from psychiatric research and give it practical meaning in daily life. I think people then begin to understand psychiatry as a field much better.

**That is the one thing I really liked it. It shows that outside of academic debates and disputes, these ideas have real-life consequences. And I have to say that there are people that I have seen a lot in Britain, and I'm sure you have them in North America, who believe that diagnoses such as ADHD are Western constructs. When you presented the research, I remember, one of them was shocked. Clearly because none of them want to put their children in those buses. What do you think of people here who dismiss these as Western constructs?**

I think that kind of thinking is part of what I call the colonial roots of global public health. Isn't it? That inherent in global health is the idea of health for the other, which is essentially an outgrowth of colonial thinking. That always the other is different from the privileged societies. But really, this ADHD study shows you what we've always known. That we are one large human family, and the neurobiology of the human race is the same, and that ADHD is a neurobiological disorder. So if you go looking for it without prejudice, you will find this in every society.

**I think this more or less sounds like colonialism in reverse. The pretence is no, we are not colonialist. But in the end, it's like a mirror image. This one does not fit in to old colonialism. But it has the same feel.**

It has the same feel.

**And to follow that, whenever I hear that phrase Western medicine, I always have the urge to retort that there's no such thing as Western medicine. There is medicine. How do you respond to this kind of phrase?**

I always tell the story of the origin of psychopharmacology; I mean modern psychopharmacology. There is a great article written by Prince, I think, in the *American Journal of Psychiatry*. In this article, he describes a Nigerian student with psychosis in London in the 1920s, who was treated with known antipsychotic herbs from Nigeria, when his father sent his healer to treat his son in London. It was the first time that Western physicians in this modern era had seen a patient with psychosis being treated medically. At that time in England, I think they were hosing patients down with cold water and using strait jackets. But in Africa, the traditional healers understood psychotic illnesses to be illnesses, and that there were specific plants that were used to treat these illnesses. And that plant, which we now know to be *Rauwolfia*, contains reserpine. Reserpine was one of the first active ingredients known to have an impact on psychotic symptoms. So as you say, this is just medicine. Knowledge and medicine is universal. It comes from different sources and from different cultural health experiences. So in West Africa, the people always knew that psychosis was a brain disease, and that indeed, there were treatments in the community that would relieve patients’ symptoms.

**Talking about colonialism reminds me of your book.^1^ I found it very interesting. And the other colleagues that I recommended the book to found it interesting also. Could you explain what made you decide to write a book about your ancestry?**

There is not a simple answer to it. I think that it was actually a process. I believe it was a process of dealing with the loss of my father. I think that's how it started. After my father died, I started sort of musing more and more about why an African like myself had a strange Dutch name. So I started thinking about it a bit more seriously and started researching it. Then a number of events occurred, and I was able to trace this family name all the way back to the 1730s, when the first Dutch person with that name arrived in Elmina,^2^ and that we are essentially descendants of this person carrying this name. But it also allowed me to look back over ten generations. The historical evolution of Ghana through the lens of a family, over ten generations. What was happening during the slave trade, what was happening after the slave trade, what was happening during the recruitment of soldiers to Java, what was happening during the independence struggle, till the present day in the post-independence period, which I call the period of the children of exile, where we are all spread around the world and struggling to find ways to be relevant back in Ghana.

**I was wondering how much your experience as a psychiatrist who goes and takes history, and as a researcher, helped you in writing this book.**

Well, one of the things that we do as psychiatrists that no other specialty in medicine does well, is that we listen very closely to people's stories, as a backdrop for everything. We are the people who really take a history. We are the listeners of stories in psychiatry. So I think, gathering that historical information for me, actually came quite naturally as a psychiatrist. And I also started projecting my own interpretations on what I was finding in the historical documents. So it was it was a very interesting process. In a way it was maybe a posthumous gift to my father to resolve the loss that I suffered with his death. And then it was also a present to my children, so that they could read about their family history for the rest of their lives.

**What made me interested was that it was a process of reconnecting with the family, which is a very good way of going through bereavement. Most things nowadays have become so individualistic. This process for you was, it seems to me, to be more meaningful than, for example, counselling.**

Yes it was. It was very cathartic. It was really a complicated way of dealing with a very important loss in my life. Yes. Because my father and I were very close.

**Finally, as a psychiatrist who is Ghanaian and has worked in the Ghana, the USA and Canada, and has actually explored things about colonialism in Ghana, what do you think of this view that we have to decolonise psychiatry.**

I mean, the mind is not a unique Western experience. The mind is a universal experience of every human being, right? So I think go back to the anecdote I gave you about the treatment of psychosis, which really shows you that within the African traditional medical context, psychiatry was not separated from other aspects of medicine. Traditional healers practised integrated health, more than we do right now in the West, where psychiatry has been removed and is separate from medicine. Which brings us back to our initial question about the curriculum at Cape Coast and the fact that what we sought to do was to integrate psychiatry closely with medicine, for the students to understand that no patient comes to you with somatic complaints and leaves their mind and emotions at home. It comes as one package to you. This is what we've tried to teach the students at this medical school. The psychiatric foundations of medicine.

## Footnotes

1 Ulzen T.P.M. Java Hill: An African Journey: A Nation's Evolution Through Ten Generations of Family Linking Four Continents. Xlibris Publishing, 2013.

2 A port in Ghana with a famous, or rather infamous, castle.

